# Chemical Characterization and Antiplatelet Potential of Bioactive Extract from Tomato Pomace (Byproduct of Tomato Paste)

**DOI:** 10.3390/nu11020456

**Published:** 2019-02-22

**Authors:** Ivan Palomo, Anibal Concha-Meyer, Mariane Lutz, Macarena Said, Bárbara Sáez, Adriana Vásquez, Eduardo Fuentes

**Affiliations:** 1 Thematic Task Force on Healthy Aging, CUECH Research Network, Universidad de Talca, Talca 3460000, Chile; ipalomo@utalca.cl (I.P.); mariane.lutz@uv.cl (M.L.); 2 Interdisciplinary Center on Aging, Thrombosis Research Center, Department of Clinical Biochemistry and Immunohematology, Faculty of Health Sciences, Universidad de Talca, Talca 3460000, Chile; 3 Centro de Estudios en Alimentos Procesados (CEAP), CONCIYT, Gore Maule, Talca 3460000, Chile; macarenasaid@gmail.com (M.S.); bsaez@ceap.cl (B.S.); 4 Faculty of Agricultural Sciences, Universidad de Talca, Talca 3460000, Chile; 5 Interdisciplinary Center for Health Studies, CIESAL, Faculty of Medicine, Universidad de Valparaíso, Angamos 655, Reñaca, Viña del Mar 2650000, Chile; 6 Faculty of Health Sciences, School of Nursing, Universidad de Talca, Talca 3460000, Chile; advasquez@utalca.cl

**Keywords:** tomato pomace, extract, platelet, tolerance, clinical pilot study

## Abstract

We examined the ability of tomato pomace extract (by-product) to affect platelet aggregation in healthy humans (clinical pilot study). In phase 1 the tolerance of participants (*n* = 15; 5 per dose level) ingesting tomato pomace extract across three dose levels (1, 2.5, and 10 g) was evaluated. Phase 2 was a single-blind, placebo-controlled, parallel design human (male, *n* = 99; 33 per group) pilot intervention trial investigating the acute and repeated dose effects (5 days) of different doses of tomato pomace extract (1 g, 2.5 g or placebo) on platelet aggregation ex vivo. Various flavonoids (coumaric acid, floridzin, floretin, procyanidin B_2_, luteolin-7-*O*-glucoside, kaempferol, and quercitin) and nucleosides (adenosine, inosine, and guanosine) were identified in the tomato pomace extract. The clinical study showed that the daily consumption of 1 g of aqueous extract of tomato pomace for 5 days exerted an inhibitory activity on platelet aggregation.

## 1. Introduction

Epidemiological evidence has shown that tomatoes and tomato based products are associated with a reduced risk of cardiovascular diseases (CVD) [1,2]. This association is supported by clinical intervention trials demonstrating the ability of the dietary intake of tomatoes and tomato based products to improve lipid profile [3,4,5], induce anti-inflammatory [5] and antiplatelet activities [6,7].

Platelet activity is accepted to play major roles in the development and progression of atherothrombosis, the main cause of CVD [8]. Modulation of platelet activity is largely achieved through prescribed pharmacological agents such as aspirin and glycoprotein inhibitors, approach used in the primary and secondary prevention of coronary events in high risk individuals [9]. However, healthy individuals (middle age and older adults), smokers, and people under stress can also exhibit impaired platelet activity. Therefore, a dietary approach to maintaining cardiovascular health may be considered another important tool in the prevention of CVD [10,11,12].

Tomato pomace (TP) constitutes about 5% of the processed tomato. It consists of tomato peel and pulp (78%) and crushed seeds (22%). TP contains fibre, sugars, proteins, lipids, vitamins, and minerals [13]. It is also a good source of flavonoids, carotenoids, and nucleosides, which have been shown to reduce platelet aggregation [14]. Indeed, in vitro, ex vivo, and in vivo assays using aqueous extracts of fresh tomatoes and TP on platelet function have demonstrated a decrease in platelet aggregation [15,16,17]. Moreover, the inhibitory effect on platelet aggregation is higher in TP extracts when compared with fresh tomato extracts [15,17]. Furthermore, to support the findings of laboratory model assays on the effect of TP on the reduction in platelet aggregation, rigorous and well controlled human intervention trials are required, since the bioactive compounds present in foods are not always the same as those found in vivo after ingestion their bioavailability may be low [18].

To our knowledge, there are no studies reporting on the effects of ingesting TP extracts on platelet aggregation in humans. The aim of this study was to determine the effects of the dietary intake of TP extracts at two different doses on platelet aggregation in adult male healthy subjects. Prior to the clinical pilot trial, a dose tolerance study to the TP extracts was conducted.

## 2. Materials and Methods 

### 2.1. Tomato Pomace Extract

The TP aqueous extract was provided by Centro de Estudios en Alimentos Procesados (CEAP, Talca, Chile). Briefly, TP was dried, ground, sonicated in water and the aqueous extract was obtained by centrifugation. For both study phases, the TP extract was ingested as a flavored drink. An orange flavored beverage powder (Zuko^®^ Light; Tresmontes Lucchetti, Valparaiso, Chile) was diluted with water according to the manufacturer’s instructions. The appropriate mass of TP extract was mixed with the flavored beverage to a final volume of 100 mL. For compliance and standardization purposes, all participants ingested the beverages within the research facility each morning for the 5 treatment days. The placebo consisted of the orange flavored beverage without the addition of TP.

### 2.2. Physical-Chemical Characterization of Tomato Pomace Extract

Soluble solids content (°Brix) was determined using a digital refractometer (HI 96801, Hanna Instruments, Woonsocket, RI, USA). pH was determined using a pH meter (HI 83141, Hanna Instruments) previously calibrated with pH 4.0 and 7.0 buffer solutions [19]. Moisture was determined in a humidity analyzer (PMR 50/1, Radwag, Radom, Poland). Water activity (a_w_) was assayed at room temperature using an aWmeter (Hygrolab C1, Rotronic Instrument Corp.; New York, NY, USA) according to AOAC (2012) [19]. Color was determined using a colorimeter (RGB-1002 Color Analyzer, Lutron, New Taipei, Taiwan) and particle size was measured with mesh screen 425 μm. Sodium, carbohydrates, protein, ashes, total sugars, total dietary fiber, fat and moisture were determined according to AOAC (2012) [19].

### 2.3. Detection and Quantification of Bioactive Compounds by High-Performance Liquid Chromatography-Mass Spectrometry (HPLC-MS)

Flavonoids, carotenoids and nucleosides contained in the TP extract were analyzed. The samples were drained in a thin mortar with 5 mL of 75% methanol and then 100 µL of internal standard (Naringenin) was added. Samples were placed in the UHPLC-MS (Thermo Scientific Exactive Plus Orbitrap Mass Spectrometer, Thermo Fisher Scientific, Bremen, Germany) autosampler rack, previously programmed for the flavonoids, nucleosides or carotenoids methodology. Mass quantification was carried out with an Exactive Plus spectrometer (Thermo Scientific, Waltham, MA, USA) equipped with an electro-spray interface operating with negative ionization mode.

### 2.4. Clinical Pilot Study

#### 2.4.1. Study Population

The study was conducted in two phases. For both, apparently healthy men ranging from 18 to 26 years old were recruited in Talca, Chile. The study period was from August 2016 to June 2017. The exclusion criteria were as follows: smoking; chronic medical conditions (e.g., gastrointestinal disease, diabetes, asthma); kidney or liver disease; medications and dietary supplements (e.g., lycopene and tomato supplements) judged to affect platelet function; clinical chemistry results at eligibility assessment judged to affect the trial outcome or be indicative of a health problem. The trial was conducted at the University of Talca and CEAP (Talca, Chile). All procedures were approved by the University of Talca Research Ethics Committee. Each participant gave written informed consent prior to taking part in the trial. The trial is registered with clinicaltrials.gov (Ref: NCT02986165).

#### 2.4.2. Study Design

Phase 1 was an escalating dose study assessing the tolerance of human participants to TP extract. For this, three dose levels (1, 2.5, and 10 g) were assayed. TP extracts were ingested during fasting in ascending order with flavored beverages, each dose level separated for a minimum of one week period. Male participants (*n* = 15; 5 per dose level) ingested TP extract once daily for 5 days. Blood pressure, pulse rate and gastro-intestinal well-being were assessed before and 3 h after ingestion of TP extract every day. In Phase 1 the effect on platelets was not evaluated. Blood samples were collected to determine the biochemical profile at the start and 24 hour (6-day) after at the end of the 5-day treatment period. Biochemical profile was determined using Automatic Spectrophotometer (Mindray BS300, Shenzhen, China) and Hematology Analyzer (Mindray BC3600, Shenzhen, China) in samples of serum and EDTA whole blood, respectively. Blood pressure and pulse rate were measured during the five days of treatment using an automatic blood pressure monitor. Clinical results and gastro-intestinal questionnaires were evaluated at the end of each TP dose level. 

Phase 2 was a single-blind, placebo-controlled, parallel human pilot intervention trial to assess the effects of acute and repeated doses of two different levels of TP extract on platelet aggregation. Male participants (*n* = 99; 33 per group) received TP extract once daily for 5 days. Given that the general lifespan of a platelet is around 10 days, the antiplatelet activity was evaluated for 5 days. Similar protocols to evaluate antiplatelet activity have been described [7,10]. For the intervention, flavored beverages containing: (i) 1 g TP extract (ii) 2.5 g TP extract or (iii) placebo (no extract). For randomization purposes, the two treatments and placebo were designated with a letter (A, B, or C), and randomization of participants to each treatment was assigned using the computer program randomization.com^®^. The participants were blinded to the treatments. For 14 days prior to the baseline assessment day and during the 5-day treatment period, the volunteers were instructed to completely exclude some foods/beverages known to affect platelet function (e.g., tomato and tomato based products, alcohol, cocoa and cocoa products, tea and coffee, among others) from the diet. To assess the acute effects of the TP treatment on platelet aggregation, blood samples were obtained before ingestion and 3 h after ingestion on days 1 and 5. The longer-term effects on platelet aggregation were assessed from the samples collected on days 1 (acute effect) and 5 before ingestion (chronic effect).

### 2.5. Platelet Aggregation Assay

To assess platelet aggregation, whole blood (12 mL) was collected directly into 3.2% sodium citrate tubes (9:1 *v*/*v*) after discarding the first 2.0 mL of the draw. Samples were centrifuged (240× *g*, 10 min) to obtain platelet-rich plasma (PRP). After aliquoting a portion of the PRP to determine platelet count the samples were centrifuged (650× *g*, 10 min) to obtain platelet-poor plasma (PPP). The PRP was adjusted to 200 × 10^9^ platelets/L with the PPP. Aliquots of adjusted PRP (480 µL) were then pre-incubated (37 °C, 3 min) before the addition of the platelet agonist adenosine diphosphate (ADP) (two final concentrations: 4 and 8 µmol/L). Platelet aggregation was measured by light transmission according to Born and Cross [20] using a Lumi-aggregometer (Chrono-Log, Havertown, PA, USA). The results of platelet aggregation (percentage maximal amplitude, area under the curve (AUC), slope, and lag time) were determined using the software AGGRO/LINK (Chrono-Log) [21]. 

### 2.6. Statistical Analysis

Phase 1 study results were analyzed using analysis of variance (ANOVA) followed by Tukey’s post hoc test. Phase 2 study was intended to determine a mean reduction in platelet aggregation of 17.4% at 90% power with a significance level of 0.05. The calculations supported the recruitment of 99 participants in total (*n* = 33 participants per group). Statistical analyses of the data were performed using the R data analysis software [22]. To assess the effect of ‘treatment’ on the outcome variables of platelet aggregation (percentage of maximal amplitude, AUC, slope, and lag time), post-pre-differences (d1 3h–d1 0h; d5 0h–d1 0h and; d5 3h–d5 0h) data were submitted to ANOVA. In addition, paired comparisons within treatments were done with the Wilcoxon paired test. Data were analyzed separately for each level of agonist concentration (4 or 8 µmol/L). Data were checked for outliers and normal diagnostics were applied (e.g., normality of model residuals). Results were considered significant if *p* < 0.05. 

## 3. Results

### 3.1. Physical-Chemical Characterization of Tomato Pomace Extract

The TP extract obtained presented low humidity (13.6 g/100 g), aW and pH, therefore it was considered microbiologically safe. The TP powder exhibited a small particle size, which aids for the utilization as an ingredient in food formulations. The chemical analysis (g/100 g) showed a protein content of 17.7, total fat 11.8, carbohydrates 30.8, total dietary fiber 21.2, ashes 7.7, and sodium 69.2 mg/100 g. 

### 3.2. Detection and Quantification of Bioactive Compounds by HPLC-MS

Table 1 shows the content of the bioactive compounds measured in TP (flavonoids, carotenoids, and nucleosides). The TP aqueous extract presented a high content of most of the detected compounds, with the exception of gallic and ferulic acids, apigenin-7-*O*-glucoside, genistein, quercitrin, daidzein, rutin, and epicatechin, which appeared in low concentrations, in some cases very close to the limit of detection. TP extract presented a high amount of nucleosides (adenosine, guanosine, and inosine). However, the results obtained for carotenoids contents indicate that the extraction methodology did not allow to concentrate these compounds. 

#### 3.2.1. Phase 1: Outcome of Safety Assessment

All fifteen participants enrolled completed the five day trial, and exhibited similar age and body mass index (BMI). Their age (mean ± standard deviation, SD) was 23 ± 2, 21 ± 2, and 22 ± 2 years old and their BMI (mean ± SD) was 23.2 ± 2.2, 24.6 ± 1.9, and 24.4 ± 1.3 kg/m^2^ for the 1, 2.5 and 10 g TP extract doses, respectively. 

At the beginning of the study (day 1—d1, time 0—0 h), all participants had similar values of systolic blood pressure (SBP), diastolic blood pressure (DBP), heart rate (HR) or biochemical profile. At the end of the period, there were no abnormal deviations in SBP, DBP, HR, or biochemical profile after ingestion of the TP beverage with any of the doses tested (Table 2 and Table 3). Two participants reported mild gastro-intestinal symptoms of discomfort after the intake of TP. Of these, the symptoms reported were mild abdominal rumbling (one case) and mild abdominal bloating (one case) when assessed 3 h after the intake of TP on days 1 and 2 of the 5-day trial (dose = 10 g). Also the 10 g TP showed solubility problems. Nevertheless, all the 3 doses of TP extract were well tolerated by the participants, and none decided to discontinue their participation in the study. 

#### 3.2.2. Phase 2: Effects of Treatment on Platelet Aggregation

**Figure 1:**
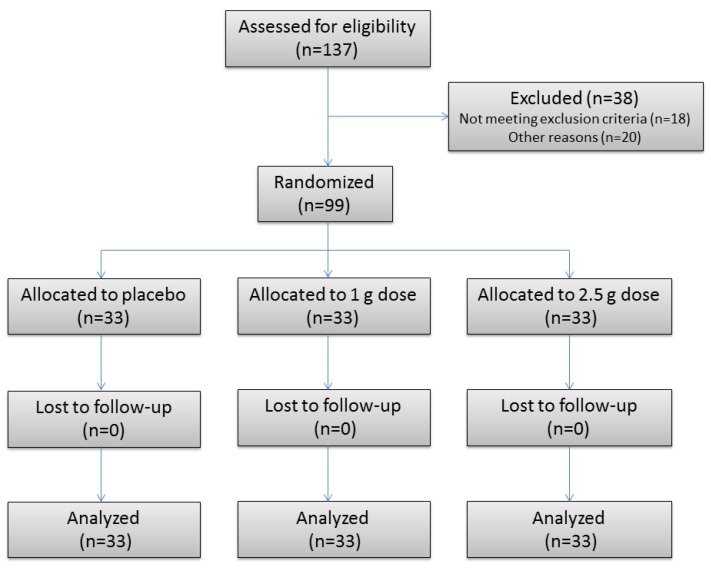
Flow of participants through phase 2 of the trial.

Of the 138 participants assessed for eligibility, 99 were randomized to treatment and completed the pilot trial. Males were selected taking into account their hormonal stability. Figure 1 shows the flow of participants through the trial. No serious adverse events were reported. The age and BMI (mean ± SD) were: 22 ± 2 years and 23.9 ± 3.0 kg/m^2^; 21 ± 2 years and 23.4 ± 2.6 kg/m^2^; 22 ± 2 years and 22.9 ± 2.5 kg/m^2^ for the placebo, 1.0 and 2.5 g TP doses, respectively.

**Figure 2:**
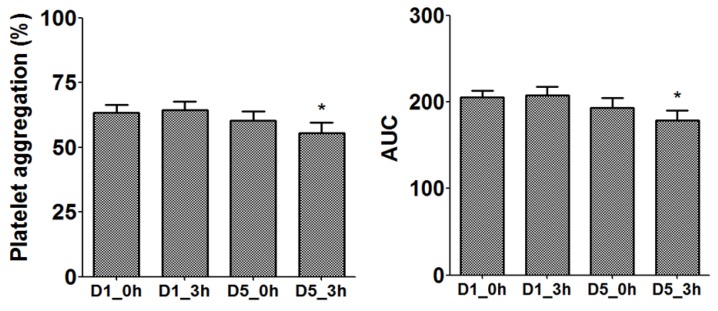
Inhibition of platelet aggregation (ADP 4 µmol/L) by 1 g tomato pomace extract. Platelet aggregation results are shown on the basis of percentage of platelet aggregation and area under the curve (AUC). The graphs depict mean ± SEM. Difference between Day 1 (d1)_0 hours (0 h) and Day 5 (d5)_3 hours (3 h) was analyzed using the Wilcoxon signed-rank test; * *p* < 0.05.

The effects of TP extract ingestion on platelet aggregation induced by ADP 4 or 8 µmol/L are shown in Table 4. The largest reduction in platelet activity was observed after the intake of 1.0 g TP during the acute phase in the 5-day treatment period (d5 0 h versus d5 3 h). Compared with placebo, the values were similar. Moreover, the analysis within groups showed that during the chronic effect only 1.0 g TP reduced the platelet aggregation induced by ADP 4 µmol/L of 62% (d1 0 h) to 54% (d5 3 h) (*p* < 0.05). This significant reduction by 1.0 g TP was observed both in percentage of maximal amplitude and AUC (Figure 2). This effect was not observed with 2.5 g TP dose or placebo.

## 4. Discussion

In this study, we hypothesized that the dietary intake of a TP extract could inhibit platelet aggregation. Although the results of parallel comparison of treatments with a suitable placebo showed no significant differences, there were significant reductions in platelet aggregation after 5 days of ingestion of 1 g TP extract. This effect was not observed both in 2.5 g dose of TP extract or placebo groups. Overall, these data suggest that there is a significant effect of the TP extract intake on platelet aggregation after repeated (5-day) dosing at a 1.0 g level of intake. 

Previous human intervention studies with tomato extract showed reductions in ADP mediated platelet aggregation of approximately 17–25% [23], while the maximum reduction observed in our study was around 9%, an effect significantly lower than that reported in other trials [6,17]. The differences between our trial and that reported by others are three-fold: (i) in some trials, subjects already known to have a high platelet response to sub-optimal concentrations of ADP were specifically selected for this phenotype. Whilst selection of specific individuals in this way reduces confounding factors influencing platelet aggregation, it could be argued that the results are not representative of the wider population; (ii) the study population in most other trials was aged 45 years and older, and although reported to be apparently healthy at eligibility. This sector of the population may be more likely to respond to the antiplatelet effects of the TP treatments; (iii) the extracts tested in other trials were minimally processed aqueous extracts of ripe tomato fruit juices (‘cold-break’ process) [6,17]. The TP used in this study was produced via a more aggressive ‘hot-break’ process which includes several heating steps that may result in losses of the specific bioactive components of the tomato fruit that have been described as modulators of platelet function [24]. However other study showed that processing and fat addition undergone by the raw tomato improved their absorption of polyphenols [25].

The results obtained demonstrate that the process of extraction of TP, assisted by ultrasound and concentration using freeze drying, allows to protect and to concentrate flavonoids of interest such as coumaric acid, floridzin, floretin, procyanidin B2, luteolin-7-*O*-glucoside, kaempferol, and quercitin. In addition, the results presented in this study are important since they show that the extraction method is effective in the concentration of nucleosides (adenosine, inosine, and guanosine). Moreover, lycopene is found mainly in tomato skin and although it is resistant to thermal processing, it is very susceptible to light [26]. In order to determine this compound, a prior extraction process must be carried out, which requires conditions of absence of light to avoid degradation, which makes its quantification very complicated and delicate. According to literature, alcohol extracts have a higher extraction yield of carotenoids than aqueous extracts, since they are lipophilic compounds that are soluble in organic solvents. Therefore, it was not expected to observe large concentrations of carotenoids in the TP aqueous extract.

The nucleosides (adenosine, inosine and guanosine) were shown to be highly active against ADP and collagen-mediated platelet aggregation [15,21,27,28]. The antiplatelet mechanism of these compounds is associated with the activation of the cAMP/PKA signaling pathway [15,21,27,28], while phenolic conjugates and flavonoid derivatives showed greater inhibition in response to thrombin and arachidonic acid mediated platelet aggregation. It has previously been reported that tomato fruit contains significant amounts of the nucleoside adenosine, and that whilst the content is markedly reduced after processing into tomato by-products such as paste and pomace, possibly because of the heat treatment steps in the manufacturing process, it does still concentration dependently reduce platelet aggregation mediated by ADP [27]. It is possible that the dose of adenosine ingested by the subjects of this study in the TP extract may have been too low to elicit an ADP mediated effect on platelet aggregation in vivo. Whilst TP is reported to retain the a significant proportion of the phenolic and flavonoid components of fresh tomato [14,29], the work conducted by O’Kennedy et al. [7] indicates that it is the least likely component of the TP to respond to ADP mediated platelet aggregation, the mode of action evaluated in the present study. Nucleosides are very water soluble and are likely to be predominantly present in the tomato juice rather than the pomace, since tomato juice accounts for approximately 95% of the original fruit [14,30]. Moreover, the aqueous extraction methodology of the TP extracts needs to be optimized in order to isolate and concentrate the main compounds responsible for platelet aggregation inhibition.

Certainly, further clinical studies are needed to correlate the consumption of TP extract with the inhibitory activity of platelet aggregation. This includes the use of lower doses of ADP and other platelet agonists, the determination of the level of oxidation of the participants and prolong the consumption time of TP extract for over 5 days, among others. 

## 5. Conclusions

This pilot clinical study concludes that the daily consumption of 1 g of aqueous extract of TP for 5 days may contribute to the inhibition of platelet aggregation.

## Figures and Tables

**Table 1:** Amounts of bioactive compounds in tomato pomace (TP) extract.

Bioactive Compound	TP Extract
Flavonoids (mg/100 g dw)	
Gallic Acid	0.83
Ferulic Acid	2.44
Coumaric Acid	88.56
Floridzin	4.71
Floretin	97.31
Procyanidin B2	1868.49
Apigenin-7-*O*-glucoside	<0.001
Kaempferol-3-*O*-glucoside	2032.58
Luteolin-7-*O*-glucoside	63.34
Genistein	<0.001
Kaempferol	77.090
Daidzein	<0.001
Quercitin	408.23
Quercitrin	1.96
Rutin	0.262
Epicatechin	0.13
Nucleosides (ug/100 g dw)	
Adenosine	42.90
Inosine	57.20
Guanosine	20.97
Carotenoids (mg/100 g dw)	
Lycopene	<0.001
Beta-carotene	55.5

**Table 2:** Changes in SBP/DBP and HR after ingestion of tomato pomace extracts across 3 different doses (Phase 1 study: tolerance).

Dose/Variable	Day 1		Day 2		Day 3		Day 4		Day 5	
	0 h	3 h	0 h	3 h	0 h	3 h	0 h	3 h	0 h	3 h
TP extract = 1.0 g										
SBP	120 ± 8	120 ± 11	113 ± 11	113 ± 7	116 ± 6	112 ± 11	117 ± 12	110 ± 7	108 ± 8	119 ± 10
DBP	74 ± 14	70 ± 8	70 ± 8	68 ± 10	71 ± 8	72 ± 4	67 ± 8	64 ± 4	64 ± 7	68 ± 11
HR	65 ± 5	69 ± 4	62 ± 4	72 ± 9	69 ± 11	74 ± 13	64 ± 8	72 ± 8	63 ± 5	69 ± 6
TP extract = 2.5 g										
SBP	110 ± 12	104 ± 10	116 ± 4	109 ± 8	118 ± 11	109 ± 8	116 ± 7	108 ± 8	112 ± 4	109 ± 3
DBP	62 ± 7	62 ± 8	68 ± 7	61 ± 2	64 ± 5	63 ± 5	64 ± 9	64 ± 4	64 ± 4	64 ± 5
HR	71 ± 11	66 ± 5	70 ± 11	70 ± 8	68 ± 5	71 ± 10	71 ± 12	73 ± 7	72 ± 7	74 ± 11
TP extract = 10 g										
SBP	110 ± 12	104 ± 6	98 ± 9	100 ± 8	104 ± 9	110 ± 9	112 ± 6	108 ± 9	111 ± 7	113 ± 11
DBP	65 ± 9	68 ± 10	64 ± 5	65 ± 5	62 ± 5	67 ± 10	67 ± 3	62 ± 6	64 ± 5	60 ± 3
HR	73 ± 5	66 ± 10	65 ± 6	67 ± 8	71 ± 6	76 ± 5	76 ± 3	79 ± 4	75 ± 5	80 ± 9

Data are Mean ± SD; *n* = 5 participants per dose level. No differences were observed between Day 1 (d1) 0 hours (0 h) and Day 5 (d5)_3 hours (3 h) analyzed using the Wilcoxon signed-rank test. TP, tomato pomace; SBP, systolic blood pressure; DBP, diastolic blood pressure; HR, heart rate.

**Table 3:** Clinical biochemistry of participants before and after ingestion of tomato pomace extract for five days across 3 different doses (Phase 1 study: tolerance).

Biochemical Parameter	TP (1 g)		TP (2.5 g)		TP (10 g)	
	Baseline	Day 6	Baseline	Day 6	Baseline	Day 6
Haemoglobin (g/dL)	15.0 ± 0.7	14.6 ± 0.7	15.3 ± 0.7	15.1 ± 0.5	15.7 ± 0.7	15.3 ± 0.7
Hematocrit (%)	45.2 ± 1.6	44.3 ± 1.8	45.6 ± 1.8	45.0 ± 1.6	47.3 ± 1.9	46.1 ± 1.5
Erythrocytes (×10^6^/µL)	5.0 ± 0.2	4.9 ± 0.3	5.2 ± 0.3	5.1 ± 0.2	5.2 ± 0.3	5.1 ± 0.1
MCHC (%)	33.1 ± 0.6	32.9 ± 0.6	33.5 ± 0.3	33.6 ± 0.6	33.2 ± 0.5	33.1 ± 0.6
MCV (fl)	89.9 ± 2.1	90.2 ± 2.1	87.4 ± 1.8	88.0 ± 1.8	89.9 ± 5.1	90.6 ± 4.6
MCH (pg)	29.7 ± 0.9	29.6 ± 1.1	29.3 ± 0.5	29.6 ± 0.9	29.8 ± 1.9	30.0 ± 1.9
Platelets (×10^3^/µL)	245 ± 55	239 ± 45	222 ± 45	222 ± 51	251 ± 89	246 ± 67
WBC (×10^3^/µL)	6.7 ± 0.8	6.7 ± 1.6	6.5 ± 1.2	6.1 ± 0.6	7.1 ± 1.5	7.2 ± 2.3
Transaminase GOT/AST (U/L)	29.0 ± 7.7	24.9 ± 2.3	20.1 ± 6.3	22.6 ± 6.3	27.4 ± 13.8	26.7 ± 6.9
Alkaline phosphatase (U/L)	101 ± 29	88 ± 19	105 ± 25	99 ± 23	105 ± 18	98 ± 18
Lactic dehydrogenase (U/L)	200 ± 81	157 ± 17	155 ± 15	155 ± 18	196 ± 62	174 ± 45
Total bilirubin (mg/dL)	1.1 ± 0.5	1.3 ± 0.9	0.9 ± 0.4	0.6 ± 0.2	0.6 ± 0.2	0.6 ± 0.2
Glycaemia (mg/dL)	83.1 ± 7.5	86.6 ± 8.5	90.7 ± 4.4	88.9 ± 5.2	85.6 ± 4.1	86.3 ± 2.7
Uric acid (mg/dL)	4.7 ± 1.6	5.0 ± 1.7	5.1 ± 0.7	4.7 ± 1.0	4.7 ± 1.7	4.5 ± 1.0
Total cholesterol (mg/dL)	141 ± 21	138 ± 18	151 ± 33	143 ± 20	170 ± 28	156 ± 27
Uric nitrogen (mg/dL)	14.7 ± 2.9	14.3 ± 3.2	16.4 ± 3.2	14.5 ± 2.9	12.6 ± 1.6	12.4 ± 5.1
Uraemia (mg/dL)	31.4 ± 6.2	30.6 ± 6.8	35.0 ± 7.0	31.0 ± 6.2	27.0 ± 3.4	26.6 ± 10.9
Calcium (mg/dL)	9.8 ± 0.2	9.6 ± 0.2	9.8 ± 0.3	9.5 ± 0.3	9.8 ± 0.1	9.7 ± 0.2
Phosphorous (mg/dL)	4.0 ± 0.5	3.7 ± 0.6	3.6 ± 0.3	3.6 ± 0.6	3.5 ± 0.5	3.3 ± 0.5
Total protein (g/dL)	7.0 ± 0.5	7.0 ± 0.3	7.0 ± 0.4	7.0 ± 0.4	7.1 ± 0.2	7.1 ± 0.3
Albumin (g/dL)	4.7 ± 0.1	4.6 ± 0.1	4.7 ± 0.2	4.6 ± 0.2	4.7 ± 0.1	4.7 ± 0.2

Data are mean ± SD. *n* = 15 participants; 5 per dose level. No differences were observed between baseline and day 6 analyzed using the Wilcoxon signed-rank test. TP: tomato pomace, MCHC: mean corpuscular hemoglobin concentration, MCV: mean corpuscular volume, MCH: mean corpuscular hemoglobin, WBC: white blood cell.

**Table 4:** Acute and chronic effects on platelet aggregation before and after ingestion of two doses of tomato pomace extract (1 and 2.5 g) and a placebo (Phase 2 study).

Dose/Time	*n*	ADP 4 µmol/L				ADP 8 µmol/L			
		Maximal Amplitude (%)	AUC	Slope	Lag Time (s)	Maximal Amplitude (%)	AUC	Slope	Lag Time (s)
**Placebo**									
d1 0h	33	68 ± 3	227 ± 12	84 ± 4	10 ± 0	79 ± 2	261 ± 9	93 ± 5	11 ± 0
d1 3h	33	70 ± 3	221 ± 10	80 ± 3	10 ± 0	82 ± 2	270 ± 10	95 ± 4	9 ± 0
d5 0h	33	67 ± 3	220 ± 10	81 ± 3	11 ± 0	83 ± 2	267 ± 8	93 ± 3	11 ± 0
d5 3h	32	65 ± 3	209 ± 9	77 ± 3	11 ± 0	80 ± 2	258 ± 8	91 ± 3	11 ± 0
**TP extract = 1.0 g**									
d1 0h	33	62 ± 3	206 ± 8	77 ± 6	11 ± 0	78 ± 2	258 ± 6	91 ± 7	11 ± 0
d1 3h	33	63 ± 3	208 ± 10	73 ± 3	11 ± 0	82 ± 2	259 ± 9	86 ± 4	10 ± 0
d5 0h	33	59 ± 3	193 ± 11	74 ± 3	12 ± 0	79 ± 3	250 ± 11	87 ± 4	12 ± 0
d5 3h	33	54 ± 4 *	179 ± 12 *	67 ± 3	12 ± 0	71 ± 4	228 ± 12	79 ± 4	12 ± 0
**TP extract = 2.5 g**									
d1 0h	33	65 ± 3	213 ± 11	81 ± 5	11 ± 0	80 ± 2	262 ± 7	97 ± 7	11 ± 0
d1 3h	31	62 ± 3	202 ± 12	74 ± 3	10 ± 0	78 ± 2	251 ± 7	84 ± 3	9 ± 0
d5 0h	33	64 ± 3	208 ± 9	76 ± 3	12 ± 0	81 ± 3	269 ± 7	91 ± 3	13 ± 0
d5 3h	32	62 ± 3	203 ± 9	77 ± 3	12 ± 0	82 ± 2	262 ± 7	88 ± 3	10 ± 0

Data are mean ± SEM. No significant differences were observed between treatments. Difference between Day 1 (d1)_0 hours (0 h) and Day 5 (d5)_3 hours (3 h) was analyzed using the Wilcoxon signed-rank test; * *p* < 0.05. TP: tomato pomace, *n*: number of participants, d: day, h: hour, AUC: area under the curve. ADP: adenosine diphosphate.
